# Management of Chronic Tinnitus and Insomnia with Repetitive Transcranial Magnetic Stimulation and Cognitive Behavioral Therapy – a Combined Approach

**DOI:** 10.3389/fpsyg.2017.00575

**Published:** 2017-04-21

**Authors:** Kneginja Richter, Jens Acker, Lence Miloseva, Lukas Peter, Günter Niklewski

**Affiliations:** ^1^University Clinic for Psychiatry and Psychotherapy, Paracelsus Private Medical UniversityNuremberg, Germany; ^2^Faculty of Social Sciences, Georg Simon Ohm University of Applied SciencesNuremberg, Germany; ^3^Faculty of Medicine Sciences, Goce Delcev UniversityŠtip, Macedonia; ^4^Clinic for Sleep Medicine ZurzachBad Zurzach, Switzerland

**Keywords:** tinnitus, insomnia, sleep, rTMS, CBT

## Abstract

It has been estimated that up to 80% of people will experience symptoms of tinnitus over the courses of their lives, with rates of comorbid sleeping problems ranging from 50 to 77%. Because of a potential connection between tinnitus and sleep disorders as well as high rates of comorbid psychiatric disorders, interdisciplinary approaches to treatment seem to be the most efficient option. In this study, we present the case of a 53-year-old male patient, who started to experience symptoms of tinnitus at the age of 49, most likely caused by work-related stress. Over the course of his illness, the patient developed comorbid insomnia. He consulted us for treatment of both conditions and we developed a treatment plan with ten sessions of repetitive transcranial magnetic stimulation (rTMS) followed by 10 sessions of cognitive behavioral therapy (CBT). We used the *Tinnitus Fragebogen* (TF) to assess the severity of the tinnitus, the Beck Depression Inventory (BDI-II) for depressive symptoms, and the WHO Well-being Index (WHO-5) for subjective well-being. Improvements could be achieved with regard to both diagnoses and the patient went from severe (48) to clinically negligible (12) TF scores, from minimal (BDI-II score 10) to no (0) depressive symptoms, and from just above critical (WHO-5 percentile 52) to above average (84) well-being. The combination of technological and psychological approaches to treat tinnitus and insomnia thus proved successful in this case. One may therefore conclude that rTMS may be considered an effective first therapeutic step for tinnitus treatment prior to CBT. To our knowledge this is the first published case in which rTMS and CBT were combined for tinnitus therapy. The approach proved successful since it led to a considerable increase in well-being and everyday functioning. To gauge the effect on a more general level, large-scale studies are still needed to cancel out potential placebo effects. Likewise, the importance of the order of the two treatments, and the possibility of using other therapies in combination with CBT to address certain tinnitus subtypes and different etiologies must be studied in greater detail.

## Introduction

When the patient first consulted us for treatment at the interdisciplinary tinnitus clinic at Klinikum Nürnberg, we performed an ear-nose-throat examination, an assessment of his psychological-psychiatric state and a basic sleep examination. These resulted in the diagnosis of an ear noise that had been chronified for 4 years and not been treated in any way yet. The patient reported that he had noticed the ear noise for the first time in connection with high pressure in his job. The ear noise made him feel helpless and caused severe difficulties to fall and stay asleep at night. He could not relax and his ability to work as well as his general well-being were severely impaired.

To deal with this situation, the patient had initially resorted to alcohol consumption in the evening as a first coping strategy (up to 7 servings of beer at a time, 3–4 days a week, at a bodyweight of 120 kg) but had already returned to abstinence when first consulting our clinic. At the time of this first examination, the patient was a non-smoker, working full time, and married.

As part of the initial assessment, we performed conventional audiometry, which showed a discrete sensorineural hearing loss in frequencies higher than 6 kHz. A cranial MRI did not show any anomalies in the patient’s brain structure. Blood work revealed no signs of anemia, decreased kidney function or abnormal levels of glucose. Two markers of liver function were slightly above normal ranges (GPT > 50 U/l at 56 U/l; gamma-GT > 55 U/l at 73 U/l), possibly due to the patient’s history of alcohol abuse. Levels of triglycerides (>150 mg/dl at 372 mg/dl) and cholesterol (>200 mg/dl at 238 mg/dl) were also increased, corresponding to the patient’s diagnosed adiposis. TSH levels were very low (<0.40 mU/l at 0.03 mU/l), indicating possible hyperthyroidism. The patient adhered to the following medication plan: Allopurinol 300 mg, Olmesartan medoxomil – Amlodipin 40 mg/5 mg combination.

The patient’s tinnitus symptoms were diagnosed as decompensated and severe according to the German tinnitus questionnaire TF (raw score 48, corresponds to percentile rank 54 and degree of severity 3; Goebel and Hiller, 1998). For tinnitus characterization, we used the TSCHQ (Landgrebe et al., 2010; Müller et al., 2016) in the German translation. The patient himself described the tinnitus as a permanent bilateral buzzing inside the head at very high frequencies, and a loudness level of 70/100 (based on a numerical rating scale from the TSCHQ). Detailed results of the assessment are presented in **Table 1**. An otoscopic examination showed normal, bilaterally intact tympanic membranes. In the Weber test, there was no lateralization at 440 Hz. A PTA test showed mild bilateral sensorineural hearing loss (right ear with a maximum of 30 dB HL at 6 kHz, left ear with a maximum of 40 dB HL at 4 kHz; assessed range: 125 Hz–8 kHz). TOAEs and DPOAEs could not be determined on a repeated basis (Supplementary Figure 1). We did not perform tone matching procedures to characterize “tinnitus pitch.”

**Table 1:** Tinnitus anamnesis by TSCHQ (Landgrebe et al., 2010; Müller et al., 2016).

Item	Answer
Age	53
Sex	Male
Handedness	Right
Tinnitus occurrence in family	No
Onset of tinnitus	4 years ago
Perceiving the onset of tinnitus	Gradual
Relation of initial onset of tinnitus	None
Pulsation of tinnitus	No
Location of tinnitus	Inside the head
Tinnitus manifestation over time	Constant
Tinnitus loudness variation from day to day	Yes
Loudness of tinnitus (1–100)	70
Description of tinnitus	Buzzing, similar to a defective fluorescent tube
Sound of tinnitus	Noise
Pitch of tinnitus	Very high
Percentage of total awake time of tinnitus awareness	90
Percentage of total awake time being distressed by tinnitus	30
Number of different tinnitus treatments	0
Reduction of tinnitus by music or environmental sounds	Yes
Worsening of tinnitus by loud noise	Do not know
Tinnitus affected by head movement or touch	No
Tinnitus affected by nap	Yes, worsening of Tinnitus
Tinnitus affected by sleep at night	Do not know
Tinnitus affected by stress	Yes, worsening of Tinnitus
Tinnitus affected by medication	No
Hearing problem	Yes
Hearing aids	No
Problems tolerating sounds	Sometimes
Sounds cause pain or physical discomfort	Yes
Headache	Yes
Vertigo or dizziness	Yes
Temporomandibular disorder	No
Neck pain	Yes
Other pain syndromes	Yes (shoulder)
Currently under treatment for psychiatric problems	Yes

In his self-anamnesis, the patient listed tension headaches up to three times a week and arthrosis in the shoulder joints. The pain caused by the arthrosis in the left shoulder was treated with conservative forms of therapy in an outpatient setting.

Regarding his sleep quality and daytime alertness, the patient stated to be experiencing considerable daytime sleepiness (Epworth Sleepiness Scale ESS = 14 – severe sleepiness, pre-therapeutic; Johns, 1991), which was aggravated by difficulties to fall and/or stay asleep at night. Here, a clear worsening could be observed over the course of time: while initially it took the patient approximately 30 min to fall asleep, this period gradually extended to times between 30 and 90 min. The patient also reported severe sleep maintenance problems, combined with early morning awakening. This meant that, in general, the patient’s overall sleeping time amounted to approximately 4.5 h. A screening with polygraphy showed habitual and at times obstructive snoring as well as a RDI of 7/h. Due to the pauses in respiration found by third-party anamnesis, a polysomnography was performed, in which an upper airway resistance syndrome was diagnosed.

In the consultation sessions, the patient made the impression to be awake, completely oriented, alert and conscious. However, a further psychological exploration showed that he was clearly suffering from inner tension, irritability and an impaired mood and a considerable decrease in drive, both in his private and professional life. The patient tried to counteract his daytime sleepiness by taking several compensatory rests and sleep breaks during the day. With regard to his cognitive and mnestic functions the psychological assessment did not show any signs of an impairment. No suicidal tendencies were observed. Regarding his general mood and possible depressive symptoms, our assessment using the BDI-II (Hautzinger et al., 2006) resulted in a raw score of 10, which corresponds to minimal depressive symptoms.

As to the patient’s ear noise, the following diagnoses were made according to ICD-10 research criteria (Dilling et al., 1994): holocephalic, decompensated and chronified Tinnitus aurium (ICD-10 H93.1) with comorbid decompensated insomnia (ICD-10 F51.0) as well as habitual and obstructive snoring (ICD-10 R06.5) and an upper airway resistance syndrome (ICD-10 G47.31). Moreover, arterial hypertonia (ICD-10 I10.0), adipositas (ICD-10 E66.0) and alcohol abuse with current abstinence (ICD 10 F10.1) were diagnosed.

## Background

Subjective tinnitus is defined as the perception of a sound in absence of an objective source (Møller, 2003). Describing its prevalence can provide a challenge, as numbers vary with age and gender (Møller, 2011). Studies postulate a lifetime prevalence of about 80% (Møller, 2011) and a point prevalence between 1 and 22.2% (Møller, 2011; Langguth et al., 2013; Kim et al., 2015; Kreuzer et al., 2016). It is assumed that between 50 and 77% of all tinnitus patients develop sleeping problems, especially those suffering from more severe forms (Crönlein et al., 2016).

With regard to effective forms of treatment, tinnitus “remains a clinical enigma” (Müller et al., 2016) due to its high levels of heterogeneity and complexity. Tinnitus may appear in various forms, regarding pattern, site, loudness or pitch of the sound, as well as other perceptual characteristics (Langguth et al., 2013). Furthermore, it can relate to several possible etiologies and comorbidities (Müller et al., 2016). A great majority of tinnitus cases do involve sensorineural hearing loss. Central auditory system reorganization is thought to be a prerequisite for chronic tinnitus.

Recent reviews (Langguth et al., 2013; Minen et al., 2014) concluded, that tinnitus may be caused by disturbances in peripheral auditory, central auditory, or non-auditory systems, by temporomandibular joint disorders, as well as by psychological factors. Regarding this last etiology, which, in our opinion, is central in this case, Mazurek et al. (2015) summarize several studies, that demonstrate associations between stress and tinnitus. According to current research, chronic tinnitus may also be triggered by muscular tension (somatosensory tinnitus), specifically in the neck (cervicogenic tinnitus) and can also appear in connection with a cervical pain syndrome, both with and without vertigo (Bechter et al., 2016). We therefore hypothesized, that a multidisciplinary, combinatory approach to treatment targeting multiple blocks could improve the treatment outcome.

When it comes to comorbid sleeping problems, reports on cases like the one discussed above suggest treating tinnitus and insomnia in conjunction: here, psychotherapy (Crönlein et al., 2007), surgery (Chen et al., 2010), as well as non-traditional approaches (Okamoto et al., 2005) may be appropriate means of therapy.

Cognitive behavioral therapy (CBT), relaxation techniques, and rTMS likewise appear to be safe and effective treatments for chronic tinnitus as they have been shown to increase the general quality of life and lower tinnitus symptoms without adverse events (Cima et al., 2012; Soleimani et al., 2016; Zenner et al., 2016). However, rTMS in particular still seems to lack the necessary methodological fine-tuning and has therfore produced moderate to large, but highly variable effects so far (Hesser et al., 2011; Langguth et al., 2013; Kreuzer et al., 2016; Soleimani et al., 2016). Other current forms of treatment were either not feasible in this case due to a lack of time and resources (e.g., notched music therapy (Okamoto et al., 2010), cochlear implantation (Olze et al., 2012) or because of their reported non to mixed-success [e.g., vagus nerve stimulation (De Ridder et al., 2014), AM-101 (van de Heyning et al., 2014), or other drugs (Kingwell, 2016)].

## Results

### Tinnitus Therapy

#### Tinnitus Therapy with rTMS

A first treatment attempt was made in October 2010. This initial treatment consisted of 10 sessions of 1 Hz rTMS over 10 consecutive working days with a stimulation intensity of 110% related to the individual resting motor threshold. 2000 stimuli per session were applied, with coil position 10–20 guided over the left primary auditory cortex.

At the time of therapy, our research group had 2 years of clinical experience in treating patients with a chronified tinnitus (Acker et al., 2010). In choosing the stimulation protocol, we followed the experiences by the interdisciplinary Regensburg working group (Kleinjung et al., 2005), who had proven a metabolic activation of the auditory cortex in a series of tinnitus patients with a similar disease process. A placebo-controlled, multicenter study including eight German participant centers on the efficacy of rTMS likewise used the treatment protocol described above (Landgrebe et al., 2008).

The primary therapeutic aim of the rTMS treatment was a favorable modulation of the patient’s tinnitus perception. An improvement in the subjective degree of severity of the symptoms could be achieved without any side effects. The main effect of the therapy was a change in the frequency and the subjective intensity of the tinnitus directly after the treatment. The initially very strong effect of the therapy decreased markedly after 3–4 h after the end of each treatment session, though. Limitations in our evaluation are the missing tone matching of tinnitus frequency and the missing high frequency audiometry (>8 kHz).

**FIGURE 1:**
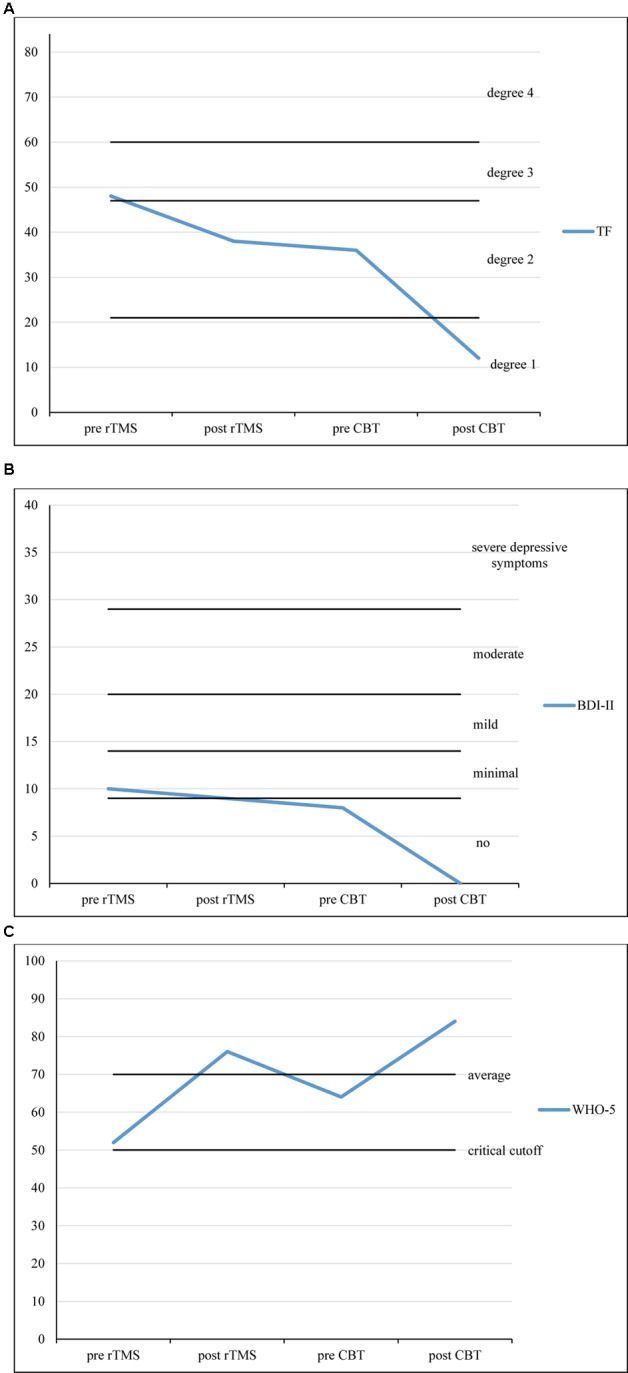
**(A)** TF scores over the course of treatment. **(B)** BDI-II scores over the course of treatment. **(C)** WHO-5 scores over the course of treatment. Timespans between measurements: pre rTMS to post rTMS: 3 weeks; post TMS to pre CBT: 2 weeks; pre CBT to post CBT: 15 weeks.

**Figures 1A–C** show the development of the patient’s raw scores across three different psychometric questionnaires (TF, BDI-II, and WHO-5) assessed at four points in time (Timespans between measurements: pre rTMS to post rTMS: 3 weeks; post TMS to pre CBT: 2 weeks; pre CBT to post CBT: 1 weeks).

The TF questionnaire is an instrument to measure tinnitus severity (α = 0.94; range: 0–84; Goebel and Hiller, 1998). Within the approximately 3 weeks of rTMS treatment, TF scores decreased from 48 (degree 3 of 4, decompensated tinnitus) to 38 (degree 2 of 4, compensated tinnitus). The BDI-II was used to measure depressive symptoms (α = 0.84–0.94; range: 0–63; Hautzinger et al., 2006). After rTMS treatment, BDI-II scores only slightly changed from 10 to 9 (minimal depressive symptoms). For the measurement of subjective well-being we used the WHO-5 (α = 0.92; range: 0–100, critical cut-off: 52, average score: 70; Brähler et al., 2007). WHO-5 scores increased from 52 (critical cut-off) to 76 (normal well-being).

#### Tinnitus Therapy with CBT

Due to the persistent professional impairment, we continued our treatment with 10 sessions of CBT as recommended by German guidelines for tinnitus management by the ADANO (Society of German-speaking Audiologists, Neurootologists, and Otologists; Lenarz, 1998) and the DGHNOKHC (German Society of Oto-Rhino-Laryngology, Head and Neck Surgery; AWMF, 2015).

The primary therapeutic aim of the behavioral therapy was to develop coping strategies to reduce the subjective impairment caused by the tinnitus as well as the resulting emotional stress. To do so, the following steps were taken: the subjective illness hypothesis was explored and discussed and medical information and explanatory models were provided. Subsequently, a connection between situative stress and tinnitus perception was worked out and, with the help of cognitive restructuring, helpful forms of behavior were successfully transferred to hitherto discouraging situations.

The patient particularly profited from practicing progressive muscle relaxation according to Jacobson as well as breathing exercises as a further relaxation method.

Within the approximately 15 weeks of CBT treatment, the patient’s TF scores decreased from 36 (degree 2 of 4, compensated tinnitus) to 12 (degree 1 of 4, compensated tinnitus). BDI-II scores had decreased to 8 (no depressive symptoms) within the 2 weeks between the last session of rTMS and the first session of CBT. They stayed at 0 after CBT treatment. WHO-5 scores increased once more, from 64 (average well-being) to 84 (above average well-being).

### Therapy of Insomnia

In view of the high subjective affliction, the patient’s sleep problems were likewise addressed with behavioral therapeutic means. Here, the main measures employed were sleep restriction and sleep hygiene (Backhaus and Riemann, 1999), as well as psychoeducation regarding sleep, cognitive restructuring of negative thoughts, stimulus control, and relaxation techniques (Piehl et al., 2010; Richter, 2015).

A polysomnography carried out subsequently found an AHI of 8/h and an increased number of respiratory arousal reactions (arousal index 15/h sleep). On diagnosis of an upper airway resistance syndrome (American Academy of Sleep Medicine, 2005) treatment with n-CPAP therapy was initiated. The snoring could be eliminated completely with average application times of 6 h/night. The patient’s compliance with CPAP treatment was very good in general.

### Further Development

After both courses of treatment (rTMS and CBT) for the tinnitus symptoms had been completed, the patient reported a notable improvement of his general state. GAF scores (global assessment of functioning; range: 0–90; Hall, 1995) increased from 45 (serious symptoms or impairment) to 68 (some mild symptoms). The ear noise and the resulting emotional distress had been reduced considerably. TF scores decreased from 48 (degree 3 of 4, decompensated tinnitus) to 12 (degree 1 of 4, compensated tinnitus). BDI-II scores decreased from 10 (minimal depressive symptoms) to 0 (no depressive symptoms). This 10-point change may be interpreted as moderate and clinically significant (Hautzinger et al., 2006). However, it must be mentioned that a placebo response to r-TMS treatment may be responsible for this change as well as intraindividual variation of subclinical BDI-scores.

WHO-5 scores increased from 52 (critical cut-off 52) to 84 (above average well-being). Likewise, there were great improvements regarding emotional balance, drive, the level of daytime sleepiness, the patient’s ability to concentrate and his general mood. Light improvements were reported concerning the ability to relax and the ability to perform.

The patient profited especially from psychoeducation on tinnitus. He could be discharged from treatment with a clearly increased self-efficacy. Throughout the entire course of treatment, the patient’s compliance was very good and co-operative. The patient also did not return to alcohol as a coping strategy but stayed abstinent throughout the entire course of treatment.

### Summary

The case of our patient demonstrates the complexity of a chronified Tinnitus aurium modulated by comorbidities. Conventional PTA showed a mild notch like sensorineural hearing loss, while high frequency hearing >8 kHz was not tested.

At the beginning of the therapy, the patient demanded a technical approach to a somatically fixated disease model. After comprehensive information on the neurological nexuses (Tyler, 2006) we agreed to a treatment attempt using rTMS. The treatment protocol follows the experiences of the Regensburg working group led by Kleinjung et al. (2007) and Langguth et al. (2015).

The psychiatric comorbidity of the present case is low for the characteristic tinnitus patient. Other working groups report a high number of afflictions from the depression – anxiety disorders range in their patient collectives (Schaaf et al., 2003; Zirke et al., 2010).

Tinnitus can have a highly negative impact on a person’s professional and private life (Zirke et al., 2013) and cause society considerable follow-up costs. Here, the severity of the tinnitus and psychiatric comorbidities are the main factors increasing costs (Maes et al., 2013). For this reason, multimodal therapy up to the point where a stability of symptoms is reached has considerable significance.

A connection between ear noises and sleeping disorders has been reported since the beginning of the 1990s (Alster et al., 1993). In some current reviews, the frequently comorbid sleep problems are conceptualized as an attendant symptom of psychiatric disorders (Mazurek et al., 2005). In contrast, other studies find insomnia-related problems in older tinnitus patients in over 50% of cases (Lasisi and Gureje, 2011). Likewise, there seems to be an increased prevalence of sleep-related respiratory disorders in tinnitus collectives (Fischer et al., 2003; Herold et al., 2010).

Regarding our patient, we hypothesize a modulation of the tinnitus perception by the insomnia understood as a hyperarousal disorder (Crönlein et al., 2016). In the presence of high subjective stress levels the interaction of auditory and limbic brain areas may be disturbed at the thalamic level, leading to a breakdown of an internal “noise-cancelation” mechanism. In this case, the activation by catastrophizing and fear seem to have played a central role (Cima et al., 2011). Potentially, there is also a bi-directional connection between tinnitus and insomnia (Rauschecker et al., 2010).

Our patient did not suffer from cervical pain or vertigo, and the tinnitus did not change after neck inclination. This lead us to exclude the cervical etiology in this case (Bechter et al., 2016).

Modern therapy concepts recommend a tailored approach in planning the therapy (Seydel et al., 2013). Similarly, for an inpatient-setting, evaluated therapy concepts are available by now (Täuber, 2012).

## Concluding Remarks

If healthcare professionals were to choose a standalone treatment for tinnitus symptoms, current evidence suggests selecting validated tinnitus-specific CBT over alternatives, such as rTMS (Zenner et al., 2016). In our case, however, rTMS therapy made it possible to start therapy at all because of a somatically fixated disease model. An optimization of the treatment protocol to improve the sustainability of the therapy seems sensible. We noticed a relevant improvement of the patient’s everyday functionality could be achieved by a stabilization of his insomnia problems. According to our assessment, a successful therapy could only be established by the combination of rTMS with tinnitus and sleep-related elements from CBT. The efficacy of this personalized combined treatment approach (Golubnitschaja and Costigliola, 2012) in larger samples will be investigated in future research.

## Ethics Statement

As all described interventions were part of clinical everyday practice, we did not consult with an ethics committee for this study. The patient gave written informed consent in accordance with the Declaration of Helsinki. The Patient also gave the consent for the analysis, processing, and publication of his data.

## Author Contributions

KR and JA contributed equally as first authors and wrote the manuscript. LM and LP contributed to the scientific design. GN contributed to data acquisition.

## Conflict of Interest Statement

The authors declare that the research was conducted in the absence of any commercial or financial relationships that could be construed as a potential conflict of interest. The reviewer KB and handling Editor declared their shared affiliation, and the handling Editor states that the process nevertheless met the standards of a fair and objective review.

## Abbreviations

ADANO: Arbeitsgemeinschaft Deutschsprachiger Audiologen, Neurootologen und Otologen German-speaking Audiologists, Neurootologists and Otologists
AHI: apnea–hypopnea index
BDI-II: Beck Depression Inventory (Hautzinger et al., 2006)
CBT: cognitive behavioral therapy
DGHNOKHC: Deutsche Gesellschaft für Hals-Nasen-Ohren-Heilkunde, Kopf- und Hals-Chirurgie German Society of Oto-Rhino-Laryngology, Head and Neck Surgery
ESS: Epworth Sleepiness Scale (Johns, 1991)
ICD-10: International Classification of Diseases (Dilling et al., 1994)
MRI: magnetic resonance imaging
n-CPAP: nasal continuous positive airway pressure
PTA: pure tone audiometry
RDI: respiratory disturbance index
rTMS: repetitive transcranial magnetic stimulation
TEOAE: transiently evoked otoacoustic emissions
TF: Tinnitus Fragebogen (Goebel and Hiller, 1998)
TSCHQ: Tinnitus Sample Case History Questionnaire (Landgrebe et al., 2010)
WHO-5: WHO-Five Well-Being Index (Brähler et al., 2007)
