# Supplementary figures and images for: Management of Chronic Tinnitus and Insomnia with Repetitive Transcranial Magnetic Stimulation and Cognitive Behavioral Therapy – a Combined Approach
Source: Front Psychol. 2017 Apr 21;8:575. doi: 10.3389/fpsyg.2017.00575 (PMC5399016; doi:10.3389/fpsyg.2017.00575)

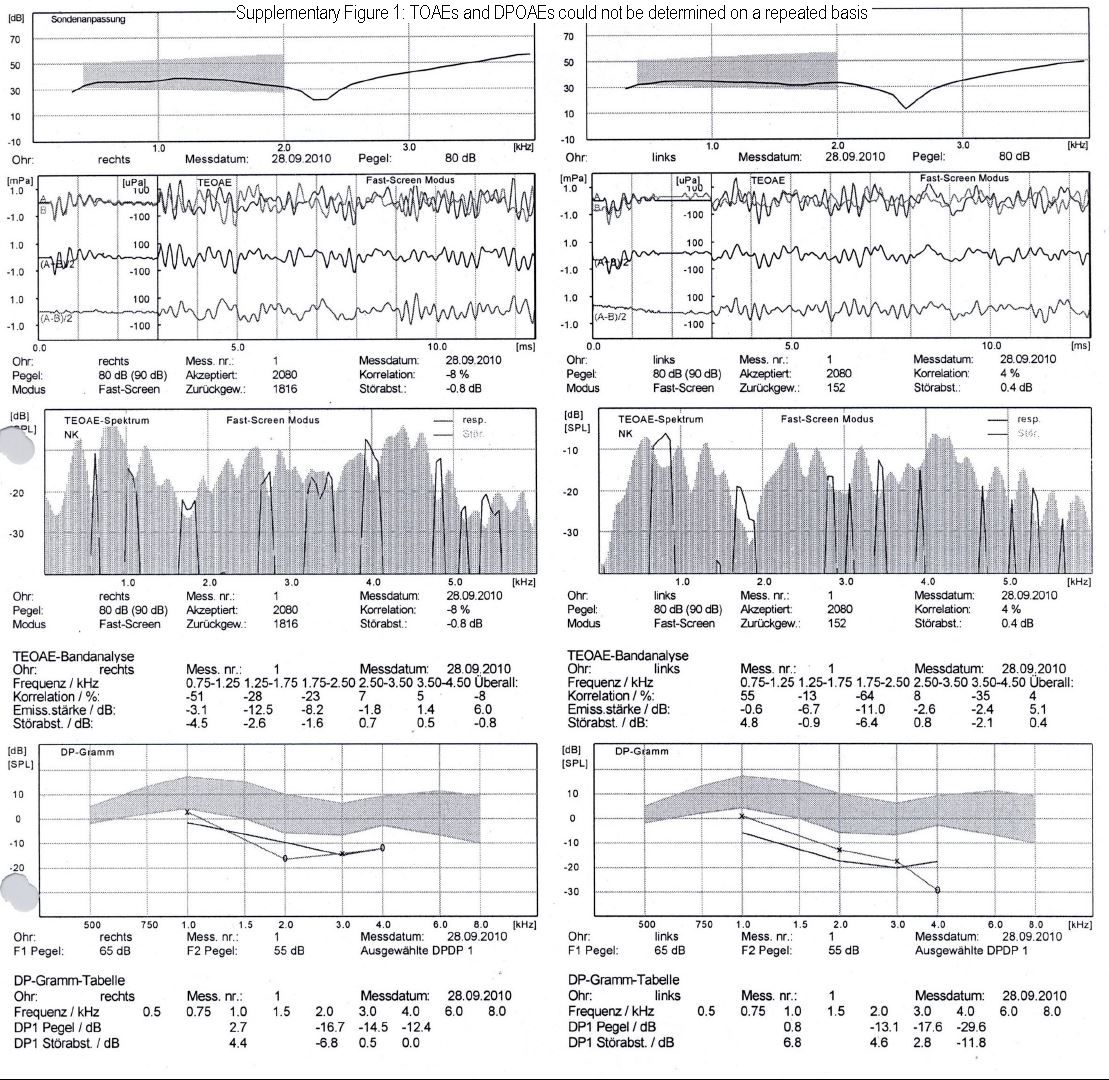

Supplement: Supplementary file 1 [file Image_1.jpg]
